# Effective Single Photodynamic Treatment of *ex Vivo* Onychomycosis Using a Multifunctional Porphyrin Photosensitizer and Green Light

**DOI:** 10.3390/jof1020138

**Published:** 2015-07-27

**Authors:** Chelsea den Hollander, Jasper Visser, Ellen de Haas, Luca Incrocci, Threes Smijs

**Affiliations:** 1 Department of Radiotherapy, Erasmus Medical Centre, P.O. Box 2040, Office Ee-1683, 3000-CA Rotterdam, The Netherlands; E-Mails: chelseadenhollander@live.nl (C.H.); j.visser.4@erasmusmc.nl (J.V.); L.Incrocci@erasmusmc.nl (L.I.); 2 Department of Dermatology and Venereology, Erasmus Medical Centre, P.O. Box 2040, Office Ee-1683, 3000-CA Rotterdam, The Netherlands; E-Mail: e.r.m.dehaas@erasmusmc.nl

**Keywords:** arthroconidia, onychomycosis, photodynamic, porphyrin, photosensitizer, trichophyton, dermatophytes, laser, LED

## Abstract

Onychomycosis is predominantly caused by the dermatophytes *Trichophyton rubrum*, *Trichophyton mentagrophytes* and *Trichophyton tonsurans.* The main treatment obstacle concerns low nail-plate drug permeability. *In vitro* antifungal photodynamic treatment (PDT) and nail penetration enhancing effectiveness have been proven for multifunctional photosensitizer 5,10,15-*tris*(4-*N*-methylpyridinium)-20-(4-(butyramido-methylcysteinyl)-hydroxyphenyl)-[21*H*,23*H*]-porphine trichloride (PORTHE). This study investigates single PORTHE green laser/LED PDT of varying degrees of *ex vivo* onychomycoses in a human nail model. *T. mentagrophytes*, *T. rubrum*, *T. tonsurans* onychomycoses were *ex vivo* induced on nail pieces at 28 °C (normal air) and 37 °C (6.4% CO_2_) during 3 to 35 days and PDTs applied to the 37 °C infections. All dermatophytes showed increasingly nail plate invasion at 37 °C between 7 and 35 days; arthroconidia were observed after 35 days for *T. mentagrophytes* and *T. tonsurans*. Using 81 J/cm^2^ (532 nm) 7-day *T. mentagrophytes* onychomycoses were cured (92%) with 80 µM PORTHE (pH 8) after 24 h propylene glycol (PG, 40%) pre-treatment and 35-day onychomycoses (52%–67%) with 24 h PORTHE (40–80 µM)/40% PG treatment (pH 5). 28 J/cm^2^ LED light (525 ± 37 nm) improved cure rates to 72%, 83% and 73% for, respectively, *T. mentagrophytus*, *T. rubrum* and *T. tonsurans* 35-day onychomycoses and to 100% after double PDT. Data indicate PDT relevance for onychomycosis.

## 1. Introduction

Onychomycosis is one of the most prevalent infections worldwide; it affects both fingernails and toenails, and its incidence is increasing [1]. The rising prevalence may be due to failure of the current therapeutics, growing number of elderly people and patients with a compromised immune system [2] or *diabetes mellitus* [3]. The dermatophytes *Trichophyton rubrum* and *Trichophyton mentagrophytes*, and to a lesser extent *Trichophyton tonsurans*, are most frequently isolated from clinical onychomycoses [4].

**Figure 1:**
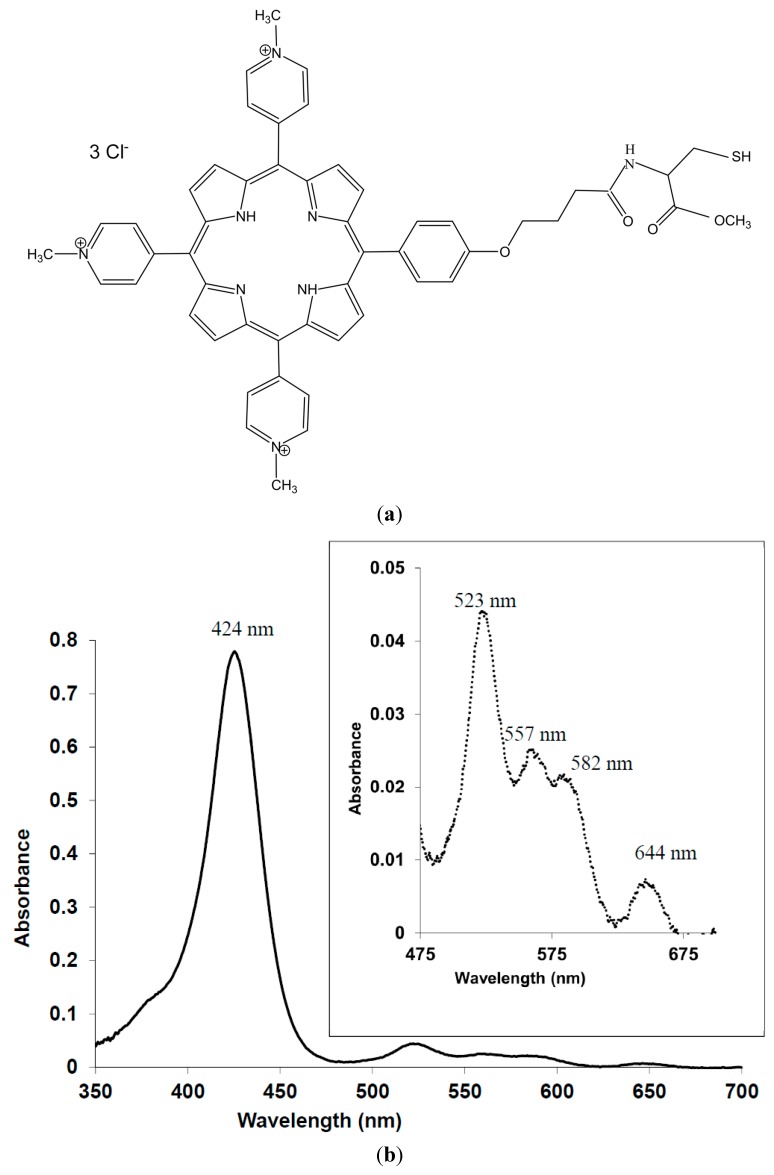
Structural formula (**a**) and representative absorption spectrum (**b**) of 5,10,15-*tris*(4-*N*-methylpyridinium)-20-(4-(butyramido-methylcysteinyl)-hydroxyphenyl)-[21*H*,23*H*]-porphine trichloride (PORTHE). The absorption spectrum was taken in 5 mM CA, pH5.

Although therapeutic options have certainly improved over the last few years, most of them still have major restrictions and a single treatment modality is not yet available. Commonly applied antifungal medications mainly act on metabolic active fungal elements thereby leaving (arthro-)conidia largely unaffected. Consequently, there is frequent recurrence of the infection resulting in long and often expensive treatments. Resistance to antifungal drugs [5,6], particularly in the case of the dermatophyte *T. rubrum* [7], is also problematic. The main obstacles for onychomycosis treatment concern the localisation of the infection in the nail plate and nail bed combined with the hardness of the nail plate and thus low permeability to drugs. These difficulties and the negative image associated with onychomycosis justify current efforts to develop new treatment modalities like photodynamic treatment (PDT) [8]. This treatment requires light-activated agents, named photosensitizers, in combination with light of a proper wavelength and, depending on the reaction type, the presence of molecular oxygen. A *type I* reaction involves an electron or hydrogen transfer between the triplet state photosensitizer and a substrate yielding ionic or free radical species, whereas a *type II* reaction involves energy transfer, ground state molecular oxygen and production of singlet oxygen (^1^O_2_) [9]. One of our recent *in vitro* studies showed the antifungal PDT (at pH 5) and nail penetration enhancing (NPE) effectiveness (best at pH 8) of a porphyrin multifunctional photosensitizer (MFPS), 5,10,15-*tris*(4-*N*-methylpyridinium)-20-(4-(butyramido-methylcysteinyl)-hydroxyphenyl)-[21*H*,23*H*]-porphine trichloride (PORTHE, Figure 1) [10]. In the present investigation, onychomycosis was induced on *ex vivo* human nails in a novel model using clinically isolated *T. mentagrophytes*, *T. rubrum* and *T. tonsurans*. Circumstances stimulating important aspects of clinical onychomycosis like fungal nail invasion and arthroconidia production were investigated. The model was therefore evaluated at different stages during nail infection and thus used to study PORTHE-mediated green laser (532 nm) and LED (525 ± 37 nm) PDT efficacy towards various degrees of *ex vivo* onychomycoses. Additionally, the influence of a known NPE compound, propane-1,2-diol (also propylene glycol, PG) on PDT effectiveness was investigated. The choice for green light was not only based on our own previous *in vitro* results with PORTHE but also on reported *T. rubrum* fungicidal effects induced by a 532 nm laser combined with rose-bengal as photosensitizer [11]. Moreover, Ghavam *et al.* recently demonstrated *in vitro* inhibition of *T. rubrum* growth using 8 J/cm^2^ 532 nm Nd:YAG laser light only [12].

## 2. Experimental Section

### 2.1. Chemicals

PORTHE (mol. wt: 987.5 g/mol; purity: 97%) was purchased from Buchem BV (Apeldoorn, The Netherlands). Photosensitizer stock solutions (8 mM) were prepared in *Milli*-Q or a 5 mM buffer solution of pH 5 (citric acid/sodium citrate, CA) or pH 8 (tris(hydroxymethyl)aminomethane, Tris) and stored at 4 °C for no longer than 2 days. All other chemicals were purchased from Sigma-Aldrich Chemie (Zwijndrecht, The Netherlands).

### 2.2. Strains

Clinical (onychomycosis) isolates of *T. mentagrophytes*, *T. rubrum* and *T. tonsurans* were kindly provided by the Department of Medical Microbiology and Infectious Diseases from Erasmus Medical Centre (Rotterdam, the Netherlands) and cultured on Sabouraud dextrose agar (SDA, Sigma Aldrich Chemie GmbH, Schnelldorf, Germany) at room temperature.

### 2.3. Nails

Healthy human nail clippings were collected and provided by pedicures (Dordrecht, The Netherlands) from anonymous sources. Additionally, the Department of Neurosciences of Erasmus Medical Centre (Rotterdam, The Netherlands) kindly allowed us to take the nails from bodies that had been donated to medical science. All nails were cleaned with 70% ethanol for 3 min in an ultrasonic water bath, stored in sealed plastic bags (room temperature) and used within 2 months.

### 2.4. Microconidia Preparation

Microconidia suspensions were prepared as described previously and stored in liquid nitrogen for no longer than 6 months [13,14]. Counting the number of colony forming units (cfu) on malt extract agar (MEA, Oxoid, Hampshire, UK) was used as a viability check.

### 2.5. Ex Vivo Onychomycosis Model

Nails, trimmed to rectangular 7–10 mg pieces, were disinfected by alternately soaking them twice in 70% isopropanol and sterile *Milli-Q*. Each nail was subsequently incubated in 100 µL. *T. mentagrophytes*, *T. rubrum* or *T. tonsurans* microconidia suspension (2.5–3 × 10^6^ cfu/mL), after 3 h transferred to a 35-mm Petri dish positioned in a 96-mm Petri dish filled with 25 mL sterile *Milli-Q* and incubated at 28 °C and normal air or 37 °C and 6.4% CO_2_ for 3 to 35 days. The CO_2_ concentration was checked weekly (Thermo Scientific G100 series AnalyserIR-CO_2_-Gastester, Geotech, London, UK). To evaluate fungal growth and arthroconidia development the nails were embedded in KP-CryoCompound (Klinipath BV, Duiven, The Netherlands) 3, 7, 14, 21 and 35 days after induction of the infection and cryosectioned (−21 °C) parallel to the surface into 10 µm slices (CM 1950 cryostat, Leica Biosystems Nussloch GmbH, Germany). Slices from the dorsal, intermediate and ventral layers were thus investigated after 20% KOH-lactophenol (25 µL lactophenol/mL 20% *w*/*v* KOH) or periodic acid-Schiff (PAS) staining. For each slice obtained from 14-, 21- and 35-day infections, 10 to 20 microscopic images were taken.

### 2.6. Light Sources

**Figure 2:**
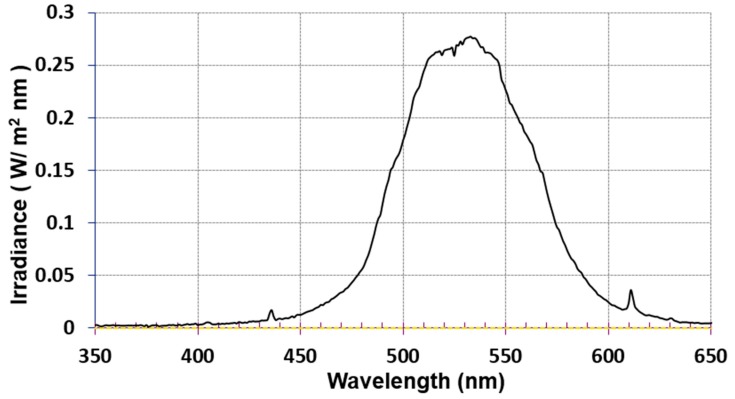
Emission spectrum of the TSPOT4-525-9 LED source showing a maximum intensity at 525 nm and a full bandwidth (measured at half the maximum intensity) of 75 nm.

PDTs were performed with a Millennia Pro s-Series diode-pumped, continuous-wave visible 532 nm laser (Spectra-Physics, Newport, CA, USA) and a TSPOT4-525-9 light emitting diode (LED, TPL vision, La Chevrolière, France).The laser power density, measured with a 2-channel power module (52961 PXI, Chroma ATE, Ede, The Netherlands) using a reversely biased photodiode (FDS02, Thorlabs, Newton, NJ, USA) on each channel, was set to 30 or 45 mW/cm^2^. The LED displayed maximum intensity at 525 nm with 75-nm bandwidth (measured at room temperature with an International Light Technologies, RPS-900-R spectroradiometer, Peabody, MA, USA, see Figure 2). The 525 nm LED power density used for the PDTs (31 mW/cm^2^) was measured with an Optometer P9710 with a 10 cm^2^ area (Gigahertz Optik, Zevenaar, The Netherlands) positioned 5.7 cm perpendicular to the incident light.

### 2.7. PDTs

**Figure 3:**
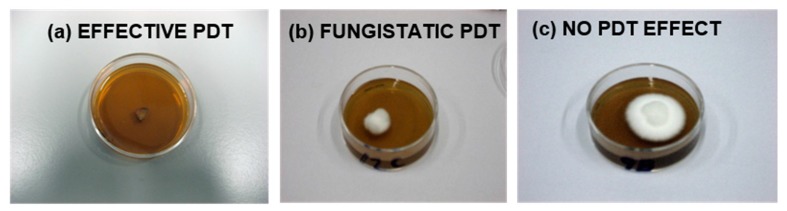
Evaluation of PDT effectiveness of *ex vivo* dermatophytic nail infections 14 days after the treatment and subsequent transfer of the nails to SDA dishes at 28 °C. PDT was considered effective when no fungal outgrowth could be detected on nail and/or SDA (**a**); fungistatic in case of a visible delay in growth compared to the growth control nails (**b**); and ineffective when fungal nail growth did not differ from that of the growth controls (**c**). Infection survival included both ineffective and fungistatic treatment efficacy. Effectively treated nail infections with no visible fungal regrowth within a 30-day follow-up period were considered cured (the situation as shown in (**a**) remains unchanged).

Laser PDT was applied to 3, 7 and 35-day-old *ex vivo T. mentagrophytes* onychomycoses while LED PDT focused on 35-day *T. mentagrophytue*, *T. rubrum* and *T. tonsurans* infections. Laser illuminations lasted 15 min (30 mW/cm^2^) or 30 min (45 mW/cm^2^) resulting in respectively 27 and 81 J/cm^2^ energy density. LED illuminations lasted 15 min (28 J/cm^2^). Prior to illumination the infected nails were incubated for 1 h in 2 mL of 5 mM CA (pH 5) or Tris (pH 8) with 40, 80 or 100 µM PORTHE on a shaker (Heidolph Rotamax 120, Schwabach, Germany). After PDT the nails were washed(*Milli*-Q), transferred to 35-mm SDA dishes, and remaining viable fungal elements allowed to grow. The treatment was considered effective in case no fungal growth could be detected macroscopically after 14 days (see Figure 3) and cured if (visible) infection recurrence failed within 30 days follow-up. In each experiment (3–5 in total), PDTs and controls were performed 6 times for each concentration and results expressed as nail infection survival percentage (compared to control). Alternatively, onychomycosis nails were pre-treated for 24 h in 40% (*v*/*v*) PG or 24 h treated with PORTHE in 40% (*v*/*v*) PG (pH 5). After the (pre-)treatment, PDT was performed as described.

### 2.8. Statistical Analysis

Statistical data analysis was performed using Pearson’s Chi-squared test, programmed in Excel with a critical level of significance of *p* = 0.05.

## 3. Results

### 3.1. Extensive ex Vivo Dermatophytic Nail Invasion at 37 °C and 6.4% CO_2_

**Figure 4:**
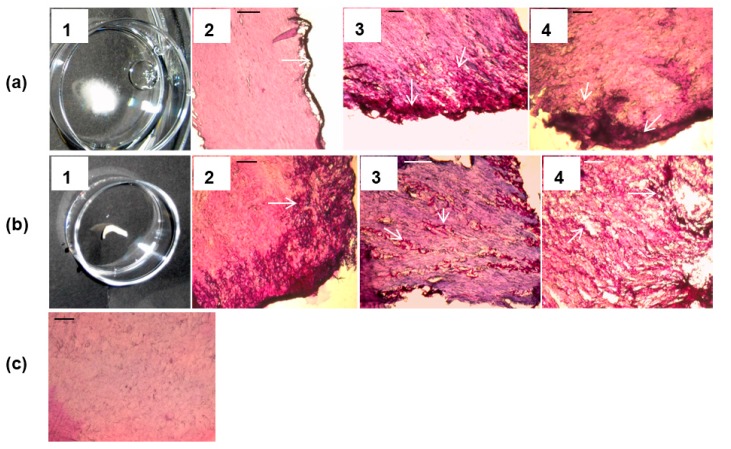
Representative images showing the growth of *T. mentagrophytus* in an *ex vivo* induced human nail infection at 28 °C (normal air, **a**) and 37 °C (6.4% CO_2_, **b**). Fourteen days after infection at 28 °C increased fungal growth was well visible outside the nail (a1) while no macroscopic outgrowth could be observed at 37 °C (b1). Representative microscopic images of PAS stained slices of dorsal (No. 2) and intermediate (No. 3 and 4) nail layers corresponding to 14 (No. 2), 21 (No. 3), and 35 (No. 4) days after *T. mentagrophytus* nail infection at 28 °C (**a**) and 37 °C (**b**) indicated increasingly fungal nail invasion at 37 °C compared to 28 °C condition. For comparison, PAS treated healthy nails incubated for 35 days at 37 °C and 6.4% CO_2_ are given in (**c**) (similar images were obtained for the 28 °C condition). Arrows indicate PAS-stained magenta fungal elements. Scale bar: 0.1 mm.

Fungal growth characteristics of 3-day-old *T. mentagrophytes* onychomycoses *ex vivo* induced at 28 °C (normal air) compared to 37 °C (6.4% CO_2_) showed macroscopically and microscopically no differences. PAS staining confirmed for both conditions fungal growth in peripheral dorsal and ventral nail zones only. Differences in growth were first noticed macroscopically for the 7-day 28 °C onychomycoses with fungal filaments starting to grow from the nails, a phenomenon that was lacking for the 37 °C infections; in this situation PAS stained cryosections indicated that fungal hyphae started to penetrate the nail. This trend continued and after 14 days at 28 °C the infections showed extensive fungal growth outside the nail (Figure 4a-1,-2), while for onychomycoses developed at 37 °C and 6.4% CO_2_, no outer fungal growth was seen (Figure 4b-1); instead, fungal nail invasion continued as indicated on PAS-stained intermediate nail sections (Figure 4b-2). After 21 days at 28 °C *T. mentagrophytes* did not penetrate the nail plate further than the peripheral zones while at 37 °C fungal elements could now be detected everywhere within the nail (compare Figure 4a-3 to Figure 4b-3). Again after 14 days, the 37 °C nail infection, prevalent in inner nail parts, started consuming the nail’s keratin (Figure 4b-4) while the 28 °C infection was still restricted to outer nail parts (Figure 4a-4). For *T. tonsurans* similar growth patterns were found while nail plate invasion of *T. rubrum* at 37 °C (6.4% CO_2_) was much faster; intermediate nail sections were reached already after approximately 14 days. For comparison Figure 4c shows intermediate nail plate slices of healthy nails incubated at 37 °C and 6.4% CO_2_. The same results were obtained for the 28 °C condition.

### 3.2. Arthroconidia Production in ex Vivo Human Nail at 37 °C and 6.4% CO_2_

**Figure 5:**
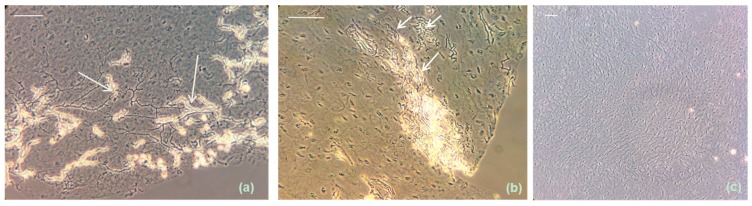
Representative images showing arthroconidia development (indicated with arrows) in *ex vivo* human nails cryosectioned and subsequently KOH-lactophenol treated 35 days after infection (37 °C, 6.4% CO_2_) with *T. tonsurans* (**a**) and *T. mentagrophytus* (**b**) compared to KOH-lactophenol treated healthy nails after 35 days incubation at 37 °C and 6.4% CO_2_ (**c**). Images were taken 4 h after KOH-lactophenol treatment. Scale bar: 0.05 mm.

Production of arthroconidia was evaluated microscopically after KOH-lactophenol treatment of cryosectioned infected nails. No conidia were detected on slices derived from the 28 °C onychomycoses. In case of the 37 °C condition arthroconidia were for *T. tonsurans* first detected approximately 21 days after the infection was induced and abundantly present after 35 days (Figure 5a). Under similar conditions *T. mentagrophytes* produced these clinically important conidiain the nails after 35 days (Figure 5b) while *T. rubrum ex vivo* onychomycoses showed arthroconidia only sporadically after 35 days. For comparison Figure 5c includes a KOH-lactophenol preparation of a healthy nail cryosection after 35 days of incubation at 37 °C and 6.4% CO_2_ showing a pattern of undisturbed onychocytes.

### 3.3. One-Time PORTHE Laser PDT (27 J/cm^2^) at pH 5 Only Effective for Non-Invasive 3-Day Onychomycoses

**Figure 6:**
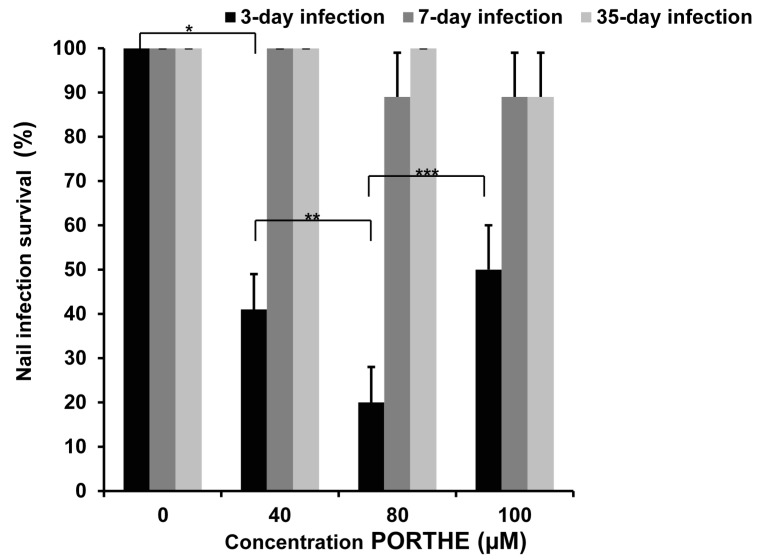
Photodynamic effects of PORTHE at pH 5 (1h incubation, 5 mM CA) towards *ex vivo* induced 3-,7-, and 35-day *T. mentagrophytes* nail infections (*ex vivo* nail model) using 15 min 532 nm laser of 30 mW/cm^2^ fluence rate (dose: 27 J/cm^2^). Presented are the mean values of nail infection survival collected from four experiments (each experiment contained 6 duplicates/concentration) and their SEM. Given survival rates remained unchanged during a 30-day follow-up period (room temperature) and effectively treated onychomycoses thus considered cured. Number of *ex vivo* fungal nail infections treated in total: 24. Dark effect: 4- to 5-day growth delay for all PORTHE concentrations for 3- and 7-day infections and 2- to 3-day delay in growth in the case of 35-day infections. *X^2^*(*) = 19.76; *X^2^*(**) = 4.46; *X^2^*(***) = 6.00. The critical value of *X^2^* (α = 0.05; *ν* = 1) is 3.84.

The 37 °C/6.4% CO_2_ combination was selected as optimal condition to induce *ex vivo* onychomycoses for the PDT experiments. As described earlier, a pH 5 formulation provides a way to selectively target PDT of dermatophytes in the presence of surrounding skin or nail by promoting selective photosensitizer binding to fungal elements instead of surrounding cells [15]. For this reason as well as our successful *in vitro* PORTHE laser PDT of *T. mentagrophytes* microconidia [10], current *ex vivo* PDT studies first focused on *T. mentagrophytes* onychomycoses, a pH 5 PORTHE formulation and 532 nm laser light. Figure 6 shows the response of 3-, 7- and 35-day old *T. mentagrophytes ex vivo* nail infections to this PDT using 30 mW/cm^2^ power density during 15 min (27 J/cm^2^). Clearly, only the infections with hardly any fungal nail invasion, namely 3-day-infections, could be successfully one-time treated with 40 and 80 µM PORTHE in 59% and 80% of the cases, respectively.

### 3.4. PG Required for Successful One-Time PORTHE Laser PDT of 7-Day and 35-Day Onychomycoses

**Figure 7:**
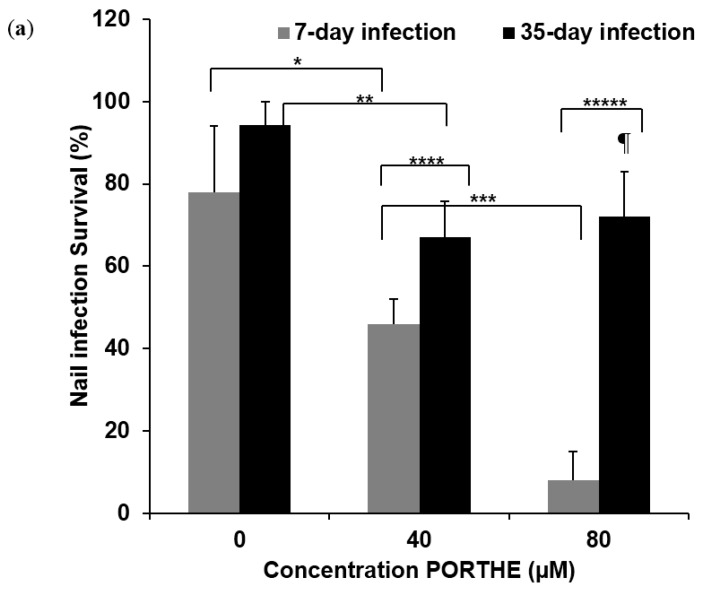

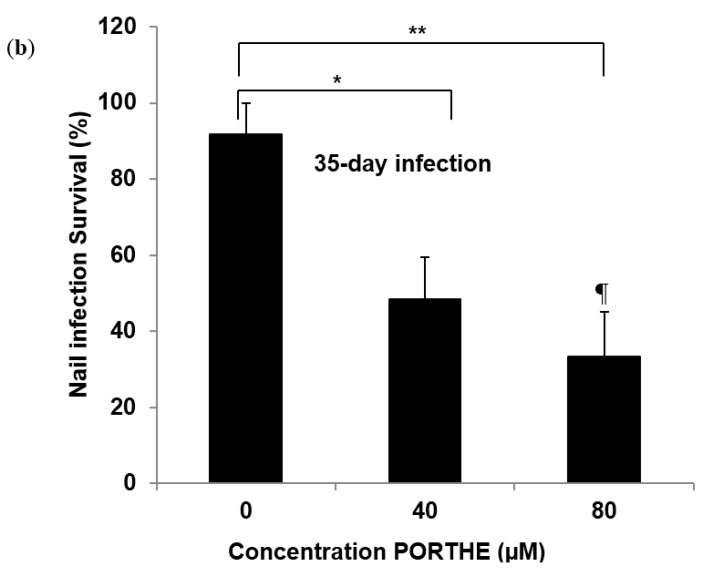
Photodynamic efficacy of PORTHE towards *ex vivo* induced 7- and 35-day *T. mentagrophytes* nail infections with 30 min 532 nm laser of 45 mW/cm^2^ fluence rate (81 J/cm^2^). Infections were pre-treated for 24 h with 40% (v/v) PG and washed with *Milli*-Q prior to PDT at pH 8 with PORTHE in 5 mM Tris (1 h incubation, (**a**)). Alternatively, the 35-day *T. mentagrophytes* infections were treated for 24 h with PORTHE in 40% (*v*/*v*) PG (pH 5) prior to PDT (**b**). Presented are the mean values of nail infection survival of four experiments (each experiment contained 6 duplicates/concentration) and their SEM. Given survival rates remained unchanged during a 30-day follow-up period (room temperature) and effectively treated onychomycoses thus considered cured. Number of *ex vivo* fungal nail infections treated in total: 24. Dark effect: an average of 10 and 15% kill in case of 7-day infections for respectively 40 and 80 µM PORTHE and 12% in case of 35-day infections for PORTHE formulated in 40% PG. A: *X^2^*(*) = 4.43; *X^2^*(**) = 4.98; *X^2^*(***) = 12.98; *X^2^*(****) = 4.68; *X^2^*(*****) = 18.29 B: *X^2^*(*) = 5.66; *X^2^*(**) = 9.07; *X^2^*(¶) = 6.22; The critical value of *X^2^* (α = 0.05; *ν* = 1) is 3.84.

To achieve a successful single PDT of 7- and 35-day increasingly invasive *T. mentagrophytes ex vivo* onychomycoses, several formulation and illumination conditions were tested, like increasing the PDT formulation pH to 8, the laser dose and a combination of these two adjustments. Certainly, PDT selectivity may well be promoted at pH 5 but the chemical reduction of keratin –S-S– disulphide bridges works best at pH 8 and higher [16]. Moreover, in our *in vitro* PORTHE studies, we also noticed that the NPE function of PORTHE worked significantly better at pH 8 compared to 5 [10]. However, incubation at pH 8 for 1 to 24 h did not increase the PDT effectiveness for these *ex vivo* onychomycoses, not even when combined with higher laser doses up to 81 J/cm^2^. Successful PORTHE mediated PDT of these 7- and 35-day infections required an additional PG treatment as well as an increased laser power and energy density of respectively 45 mW/cm^2^ and 81 J/cm^2^. Regarding the PG, a 24 h pre-treatment with 40% PG prior to PDT was sufficiently effective for the 7-day infections (see Figure 7a) while the 35-day infections needed one 24 h treatment of PORTHE formulated in 40% PG prior to illumination (Figure 7b). Ninety-two percent of the 7-day infections could now be cured with 80 µM PORTHE, a significantly higher percentage than found for the 35-day onychomycoses with the 24 h PG pre-treatment (28%, Figure 7a). Similar light conditions applied to PDT at pH 5 did not result in this improved efficacy. The PDT efficiency of 35-day onychomycoses was increased by combining PG with PORTHE treatment prior to illumination. Even though the pH of this PORTHE/PG formulation consequently dropped to 5 again significantly less 35-day infections (33%) now survived the PDT with the same 81 J/cm^2^ light dose (Figure 7b). Light and dark 40% PG controls also displayed 5 to 20% fungicidal effectiveness without infection reoccurrence (30 days follow-up). Notice that this fungicidal capacity of glycols dates back to a distant past [17,18].

### 3.5. Equally Effective One-Time LED (28 J/cm^2^) PDT of 35-Day T. Mentagrophytes, T. Rubrum and T. Tonsurans Onychomycoses with pH 5 PORTHE/PG Formulation

**Figure 8:**
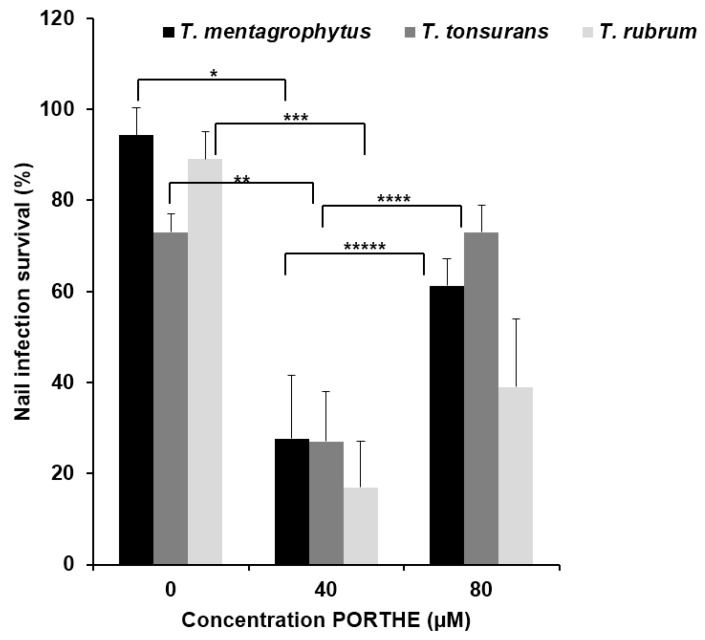
Photodynamic efficiency of PORTHE/40% (*v*/*v*) PG formulation (pH 5, 24 h incubation) towards 35-day *T. mentagrophytus*, *T. tonsurans* and *T. rubrum ex vivo* induced onychomycoses using a 525 (±37 nm) LED with power density of 31 mW/cm^2^ (energy density: 28 J/cm^2^). Presented are the mean values of nail infection survival of four to five experiments (each experiment contained 6 duplicates/concentration) and their SEM. Given survival rates remained unchanged during a 30-day follow-up period (room temperature) and effectively treated onychomycoses thus considered cured. Repetition of the complete PDT directly after the first one resulted for all dermatophytic infections in zero infection survival without any recurrence during the 30-day follow-up period (dark toxicity: 28 to 33%). Number of *ex vivo* fungal nail infections treated in total varied from 24 for *T. rubrum* and 30 for *T. mentagrophytus and T. tonsurans.* Dark effect: approximately 10 to12% kill for respectively 40 and 80 µM PORTHE/PG formulation. *X^2^*(*) = 20.35; *X^2^*(**) = 28.90; *X^2^*(***) = 25.08; *X^2^*(****) = 8.15; *X^2^*(*****) = 4.09. The critical value of *X^2^* (α = 0.05; *ν* = 1) is 3.84.

To investigate the clinical usefulness of PORTHE PDT, the light source and wavelength were optimized and the susceptibility of *T. rubrum* and *T. tonsurans* to this treatment was studied. With PORTHE having a Q-4 absorption band at 523 nm, the TSPOT4-525-9 LED (see Figure 2) seemed a good choice. These LED PDT results are shown in Figure 8 and indicate that there is no difference in susceptibility of the various dermatophytic onychomycoses with 40 µM PORTHE/40% PG and 28 J/cm^2^ light dose. On average only 24% of the 35-day nail infections survived this PDT. Interestingly, the PDT efficacy of the 80 µM PORTHE formulation decreased compared to the effect obtained for 40 µM. Figure 7a,b also shows this phenomenon (equal or decreased efficiency at 80 compared to 40 µM PORTHE) for PDT of 35-day onychomycoses. When the incubation of the nail infections with 40 µM PORTHE/40% PG was repeated directly after the illumination and followed by a second similar 28 J/cm^2^ LED dose, none of the infections survived this double PDT and an infection recurrence during a 30-day follow-up period could not be observed. However, the dark toxicity also increased to 28% to 33%.

## 4. Discussion

This study reports on one of our MFPSs designed for PDT of dermatophytic onychomycoses. With respect to the effectiveness of different onychomycosis treatment strategies, various models have been developed recently [19,20,21]. Although many of these models use a realistic topical drug application, they have done so on bovine hoof as a keratin source instead of human nail material [20,21]. The nail penetration behavior, however, will also be affected by the presence of fungal growth. On the other hand, the model used by Mehra *et al.* [19] included *T. rubrum-*infected human keratin but obtained this material from powdered nails. This implies that one of the onychomycosis treatment obstacles, the stiffness of the nail plate, cannot be tested in this model. In contrast to these recently published onychomycosis models, the model developed for this study focused on two aspects of clinical onychomycosis: fungal invasion into nail keratin and arthroconidia development. These conidia are only produced *in vitro* under specific growth conditions [22]. Our 37 °C /6.4% CO_2_ model condition met these requirements for all tested dermatophytes. The fact that *T. rubrum* arthroconidia could only sporadically be detected in nails while abundantly present under similar circumstances for *T. tonsurans* and *T. mentagrophytes* may be caused by the faster growth rate of *T. rubrum* in the model compared to the other two dermatophytes. Arthroconidia may then well be visible for only a relatively short period just before germination. Notice that arthroconidia production reached its climax only after 35 infection days, a period after which the nail is gradually consumed by the fungus in contrast to the clinical situation where the nail’s keratin is constantly renewed. The use of pH 5, a condition promoting PORTHE binding to fungal elements rather than the nail and thus improving PDT efficiency, was indeed effective when PORTHE NPE capacities were not involved like in the 3-day infections. In these cases infections were restricted to the periphery of the nails and only MFPS’ photosensitizing ability was required for overall effectiveness. As soon as the infection penetrates the nail the NPE function of PORTHE at pH 8 could be required as well. However, despite this basic pH, successful PDT of growing invasive 7-day infections required an additional chemical enhancer and increased laser dose (81 J/cm^2^). The positive effects thus obtained were not found when PORTHE was replaced by our frequently reported Sylsens B photosensitizer (unpublished results). This porphyrin is structurally related to PORTHE to the extent that it also possesses three positive charges but lacks the disulphide reducing capacity of a –SH group as present in PORTHE [10,15,23]. This may indicate that specific PORTHE multifunctional capacities, particularly the NPE function, could be partly responsible for the treatment effectivity. Differences in PDT mechanism may also play a role. *In vitro* PDT action of PORTHE was proven to pass via *type I* reactions [10] while Sylsens B mainly acts via a *type II* PDT mechanism [15]. The hardest to treat were undoubtedly invasive 35-day onychomycoses. Interestingly, the combined PORTHE/40% PG formulation resulted in high cure percentages for laser and LED PDTs. Apparently, the NPE contribution of PG to this pH 5 formulation was critical and surpassing the lower NPE effect of PORTHE at this pH, thereby favouring its PDT capacity. Although the LED (28 J/cm^2^) PDT effectiveness of this formulation for 35-day *T. mentagrophytes* onychomycoses (Figure 8) did not significantly (*X^2^* = 2.34) improve the 81 J/cm^2^ laser PDT efficacy (Figure 7b), a low-dose LED application is certainly more attractive for patients. As already remarked, both LED and laser PDT of 35-day onychomycoses always displayed equal or less effectiveness for 80 µM compared to 40 µM PORTHE. The presence of growing increase of fungus compared to nail material and similarly increasing number of PORTHE fungal binding sites could have caused this effect. High 80 µM concentration could lead to an extremely dark-coloured shield surrounding the many fungal elements and thus obstruct light penetration. Likewise, this PDT activity-decreasing phenomenon was previously found for high methylene blue concentrations [24], and in this study was found for 100 µM PORTHE (Figure 6). Consequently, the PORTHE NPE function will be less active and additional PG action required. Although our invasive *ex vivo T. mentagrophytes*, *T. rubrum* and *T. tonsurans* modelled onychomycoses could be completely cured after sequentially similar PORTHE/PG-mediated 525 nm LED PDTs, treatment of clinical onychomycosis may require additional handlings. This may relate to the differences in drug accessibility for the dorsal compared to ventral site of the nail plate. The dorsal site is often less accessible to drugs, and to solve this problem the upper site of the nail plate is currently often filed prior to any treatment. To fully access all parts of severe *in vivo* onychomycoses, our LED PDT may also require longer PORTHE/PG incubation times. Nevertheless, the presented results combined with the first PORTHE results of a single-dose dermal local tolerance study in Göttingen minipigs recently performed for us by WIL Research Europe B.V. (den Bosch, the Netherlands) may pave the way for further pre-clinical studies. According to this study, PORTHE formulated in 40% PG, in presence and absence of 28 J/cm^2^ LED light, did not result in clinical signs or irritation at dermal application sites, and PORTHE was detected on and in the stratum corneum without penetrating the epidermal cells underneath [25].

## 5. Conclusions

These data indicate that PDT may be relevant for onychomycosis. Particularly, the use of green LED light in combination to the described multifunctional porphyrin photosensitizer. This PORTHE porphyrin formulated in PG will guarantee sufficient PDT and NPE effectiveness.

## References

[1] Grover C., Khurana A. (2012). Onychomycosis: Newer insights in pathogenesis and diagnosis. Indian J. Dermatol. Venereol. Leprol..

[2] Ramos-E-Silva M., Lima C.M., Schechtman R.C., Trope B.M., Carneiro S. (2010). Superficial mycoses in immunodepressed patients (AIDS). Clin. Dermatol..

[3] Gupta A.K., Humke S. (2000). The prevalence and management of onychomycosis in diabetic patients. Eur. J Dermatol..

[4] Nazar J.R., Gerosa P.E., Diaz O.A. (2012). Onychomycoses: Epidemiology, causative agents and assessment of diagnostic laboratory methods. Rev. Argent Microbiol..

[5] Martinez-Rossi N.M., Peres N.T., Rossi A. (2008). Antifungal resistance mechanisms in dermatophytes. Mycopathologia.

[6] Kathiravan M.K., Salake A.B., Chothe A.S., Dudhe P.B., Watode R.P., Mukta M.S., Gadhwe S. (2012). The biology and chemistry of antifungal agents: A review. Bioorg. Med. Chem..

[7] Mukherjee P.K., Leidich S.D., Isham N., Leitner I., Ryder N.S., Ghannoum M.A. (2003). Clinical Trichophyton rubrum strain exhibiting primary resistance to terbinafine. Antimicrob. Agents Chemother..

[8] Smijs T.G., Pavel S. (2011). The susceptibility of dermatophytes to photodynamic treatment with special focus on Trichophyton rubrum. Photochem. Photobiol..

[9] Rajesh S., Koshi E., Philip K., Mohan A. (2011). Antimicrobial photodynamic therapy: An overview. J. Indian Soc. Periodontol..

[10] Smijs T., Dame Z., de Haas E.R., Aans J.B., Pavel S., Sterenborg H. (2013). Photodynamic and Nail Penetration Enhancing Effects of Novel Multifunctional Photosensitizers Designed for The Treatment of Onychomycosis. Photochem. Photobiol..

[11] Cronin L., Moffitt M., Mawad D., Morton O.C., Lauto A., Stack C. (2012). An *in vitro* study of the photodynamic effect of rose bengal on trichophyton rubrum. J. Biophotonics..

[12] Ghavam S.A., Aref S., Mohajerani E., Shidfar M.R., Moravvej H. (2015). Laser irradiation on growth of trichophyton rubrum: An *in vitro* study. J. Lasers Med. Sci..

[13] Zurita J., Hay R.J. (1987). Adherence of dermatophyte microconidia and arthroconidia to human keratinocytes *in vitro*. J. Invest Dermatol..

[14] Smijs T.G., Bouwstra J.A., Schuitmaker H.J., Talebi M., Pavel S. (2007). A novel *ex vivo* skin model to study the susceptibility of the dermatophyte Trichophyton rubrum to photodynamic treatment in different growth phases. J. Antimicrob. Chemother..

[15] Smijs T.G., Bouwstra J.A., Talebi M., Pavel S. (2007). Investigation of conditions involved in the susceptibility of the dermatophyte Trichophyton rubrum to photodynamic treatment. J. Antimicrob. Chemother..

[16] Ellman G.L. (1958). A colorimetric method for determining low concentrations of mercaptans. Arch. Biochem. Biophys..

[17] Mellody M., Bigg E. (1946). The fungicidal action of triethylene glycol. J. Infect. Dis..

[18] Kinnunen T., Koskela M. (1991). Antibacterial and antifungal properties of propylene glycol, hexylene glycol, and 1,3-butylene glycol *in vitro*. Acta Derm. Venereol..

[19] Mehra T., Schaller M., Walker B., Braunsdorf C., Mailander-Sanchez D., Schynowski F., Hahn R., Rocken M., Koberle M., Borelli C. (2015). Efficacy of antifungal PACT in an *in vitro* model of onychomycosis. J. Eur. Acad. Dermatol. Venereol..

[20] Tauber A., Muller-Goymann C.C. (2015). *In vitro* permeation and penetration of ciclopirox olamine from poloxamer 407-based formulations-comparison of isolated human stratum corneum, bovine hoof plates and keratin films. Int. J. Pharm..

[21] Sleven R., Lanckacker E., Boulet G., Delputte P., Maes L., Cos P. (2015). Development of a novel *in vitro* onychomycosis model for the evaluation of topical antifungal activity. J. Microbiol. Methods.

[22] Gupta A.K., Ahmad I., Porretta M., Summerbell R.C. (2003). Arthroconidial formation in Trichophyton raubitschekii. Mycoses.

[23] Smijs T.G., van der Haas R.N., Lugtenburg J., Liu Y., de Jong R.L., Schuitmaker H.J. (2004). Photodynamic treatment of the dermatophyte Trichophyton rubrum and its microconidia with porphyrin photosensitizers. Photochem. Photobiol..

[24] Ragas X., Dai T., Tegos G.P., Agut M., Nonell S., Hamblin M.R. (2010). Photodynamic inactivation of Acinetobacter baumannii using phenothiazinium dyes: *In vitro* and *in vivo* studies. Lasers Surg. Med..

[25] Lourens N. (2015). Single Dose Local Tolerance Study with PORTHE in Göttingen Minipigs.

